# Initiation of Glucagon-Like Peptide-1 Receptor Agonist Therapy and Lifestyle Modifications for Morbid Obesity in a Patient With Paroxysmal Atrial Fibrillation, Severe Osteoarthritis, and Obstructive Sleep Apnea: A Case Report

**DOI:** 10.7759/cureus.116307

**Published:** 2026-09-15

**Authors:** Brian Morris

**Affiliations:** 1 Medicine, University of California, Los Angeles (UCLA), Los Angeles, USA

**Keywords:** glp-1 agonist, glucagon-like-peptide-1, habit making, medical weight loss, weight loss and behavior, weight reduction

## Abstract

This case report describes a 57-year-old man with morbid obesity, paroxysmal atrial fibrillation, and severe knee osteoarthritis. We discuss the successful optimization of his metabolic and orthopedic risks through the initiation of a glucagon-like peptide-1 (GLP-1) receptor agonist in conjunction with a structured lifestyle modification plan. This case demonstrates the measurable benefit of combining advanced pharmacotherapy with targeted behavioral interventions to manage complex, obesity-related comorbidities. Through the course of a 12-month monitoring period, this patient shifted his body mass index (BMI) from stage 2 to stage 1 obesity, moved his blood pressure to a healthier range, and achieved measurable improvements in his lipid profile.

## Introduction

Managing morbid obesity in patients who present with multisystem comorbidities presents a significant clinical challenge. While the introduction of glucagon-like peptide-1 (GLP-1) receptor agonists to clinical care over the last decade has advanced treatment for these patients, especially related to cardiovascular risk, integrating structured lifestyle modifications remains essential to optimize patient outcomes. In this case report, we discuss clinically reproducible methods to achieve long-term health improvements through the combination of GLP-1 receptor agonist therapy in concert with a behavioral modification plan. This case discusses the clinical utility of combining advanced pharmacotherapy with targeted behavioral interventions to manage complex, obesity-related comorbidities to support long-term health gains.

## Case presentation

A 57-year-old male patient presented to the clinic for a routine follow-up appointment regarding multiple chronic metabolic and orthopedic conditions. The patient’s medical history was significant for morbid obesity, paroxysmal atrial fibrillation, hyperlipidemia, hepatic steatosis, hypogonadism, degenerative joint disease, and obstructive sleep apnea compliant with continuous positive airway pressure (CPAP) therapy. Current maintenance medications included multiple antihypertensive agents, a high-dose statin, and ezetimibe.

Upon physical examination, vital signs revealed a height of 6 feet 0.5 inches and a weight of 284.5 pounds, yielding a body mass index (BMI) of 38.1 kg/m². Blood pressure was elevated at 131/85 mmHg, with a regular pulse of 78 beats per minute. Musculoskeletal examination revealed severe bilateral knee osteoarthritis expected to require bilateral total knee arthroplasties within the next several years.

Given the risks of the patient’s cardiovascular condition and severe orthopedic condition, medical weight management was initiated by prescribing a GLP-1 agonist. Treatment with tirzepatide was initiated at 2.5 mg subcutaneously once weekly. Over the monitoring period, the dosing was increased to 5 mg, 7.5 mg, and then 10 mg with no reported side effects. Other than tirzepatide, no changes were made to the patient’s pharmacological management during the monitoring period. Concurrently, structured clinical counseling was provided, focusing on an objective, four-step behavioral modification plan to facilitate sustainable lifestyle habit change to support long-term weight reduction goals.

Over the course of the 12-month monitoring period, the combination of the introduction of a GLP-1 receptor agonist and dietary changes resulted in this patient achieving clinically significant improvements in his low-density lipoprotein (LDL) and triglyceride levels (Figures 1, 2).

**Figure 1:**
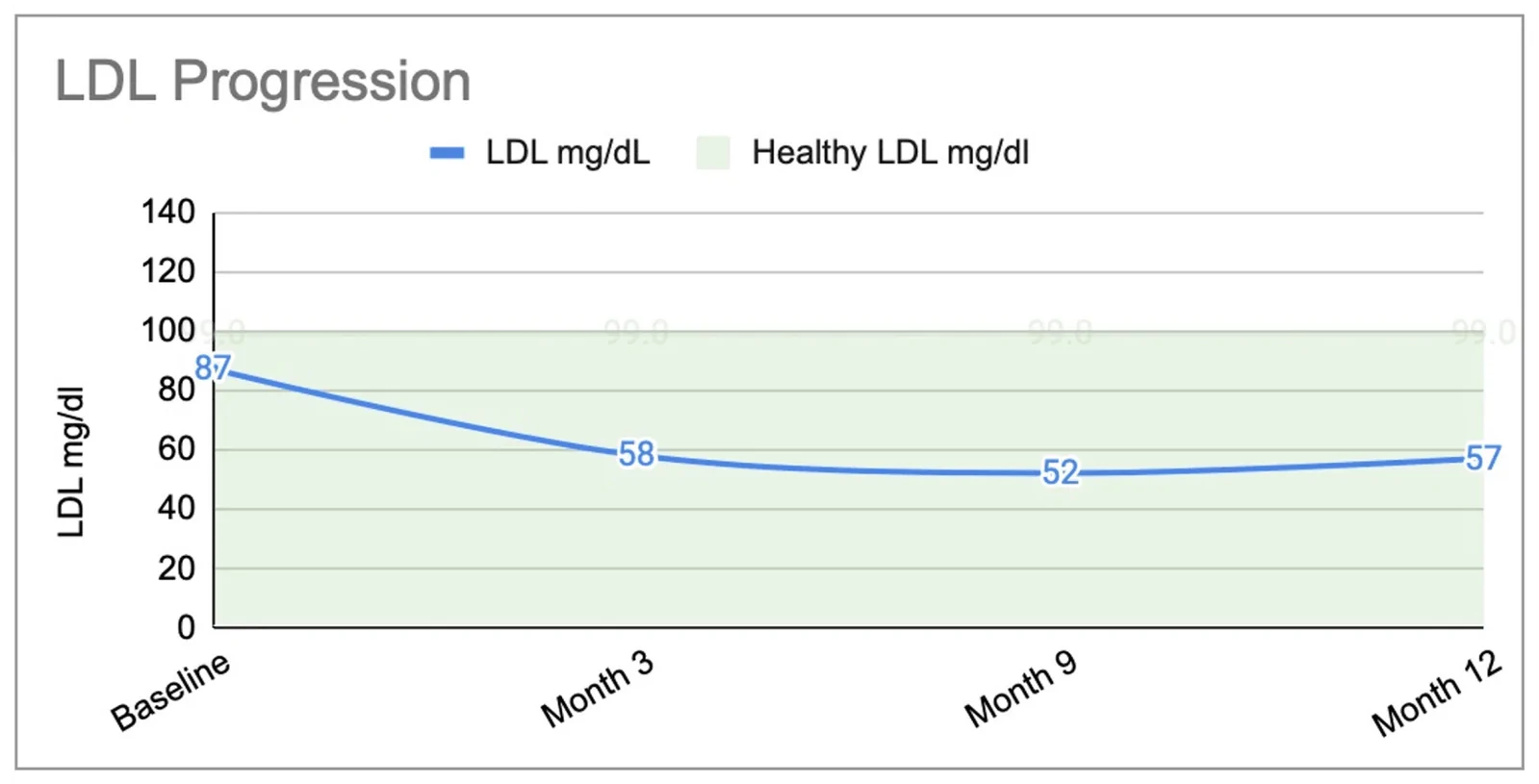
Progression of low-density lipoprotein (LDL) over monitoring period

**Figure 2:**
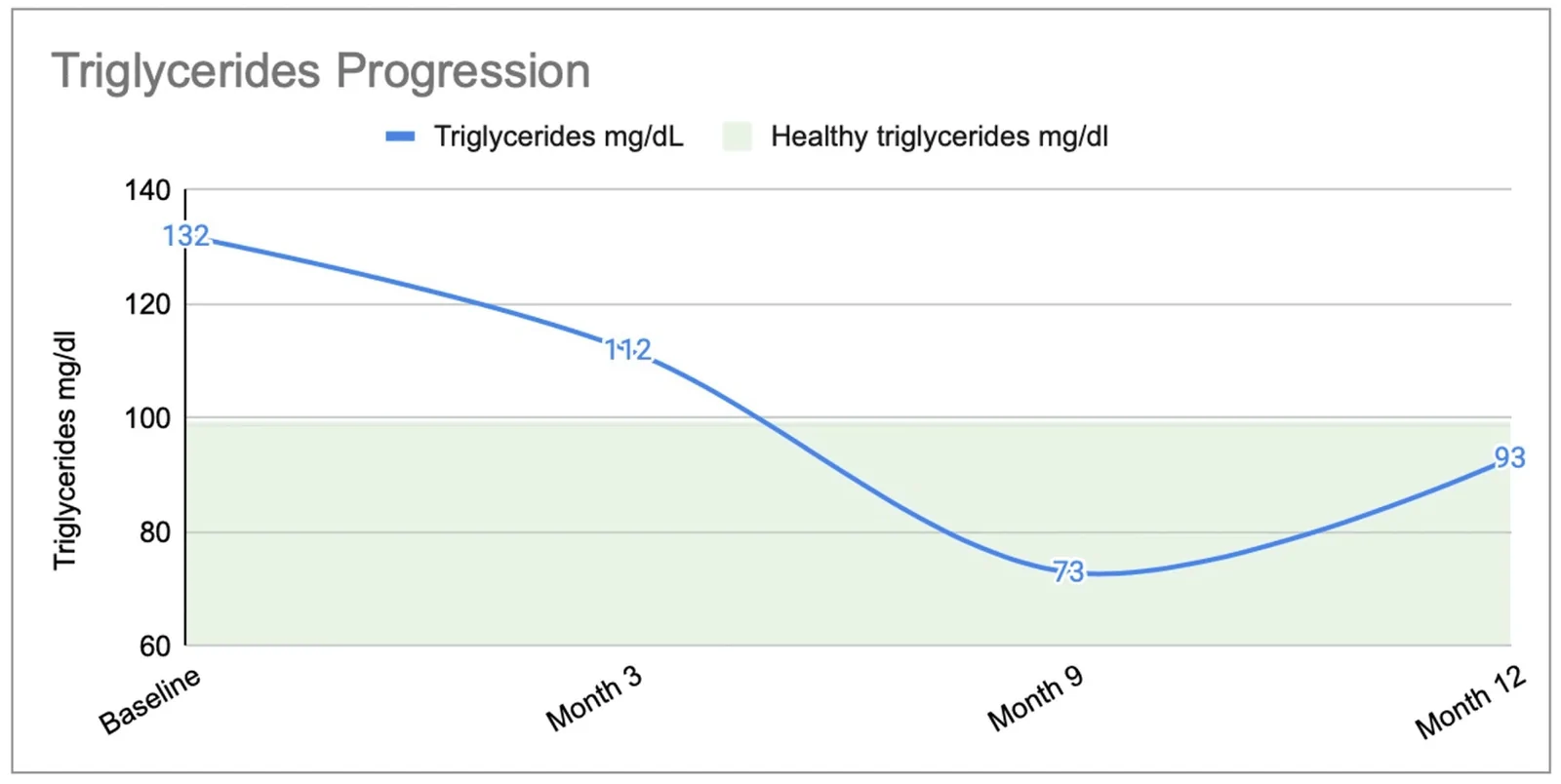
Progression of triglycerides over the monitoring period

By the end of the 12-month monitoring period, the combination of weight loss, achieved through pharmacotherapy and behavioral modification, along with stress-reduction techniques taught in clinic, resulted in a lowering of the patient’s systolic blood pressure with a concomitant stability in his diastolic blood pressure (Figure 3).

**Figure 3:**
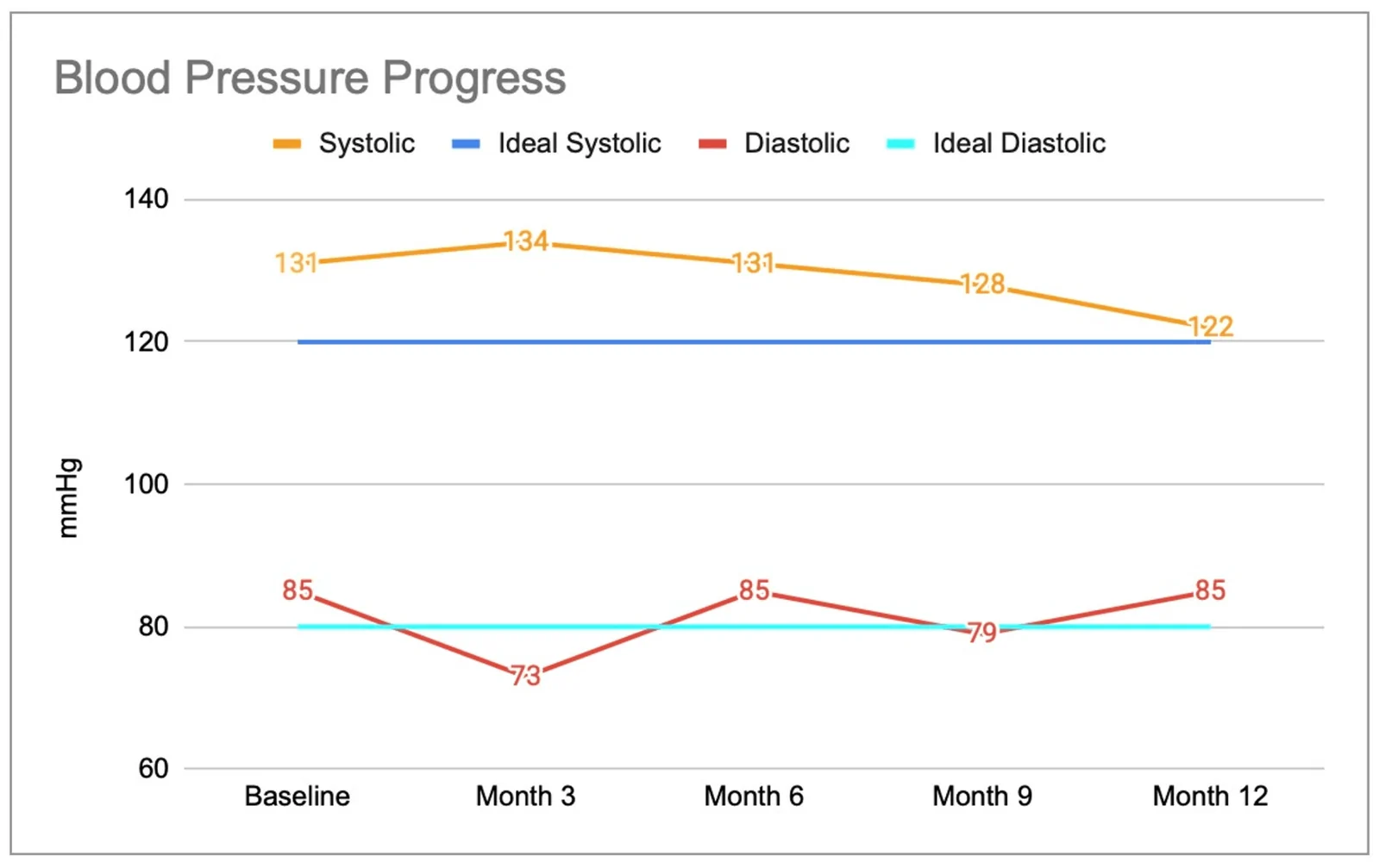
Progression of systolic and diastolic blood pressure over the monitoring period

This patient also experienced significant weight loss over the monitoring period. Through the use of a GLP-1 receptor agonist in conjunction with a highly personalized lifestyle modification framework, this patient achieved quantifiable health improvements. Notably, this 57-year-old man reached a weight of 238 pounds, which represents a reduction in BMI from 38.1 kg/m² to 31.8 kg/m² and shifts this patient from obesity class 2 to class 1 (Figure 4).

**Figure 4:**
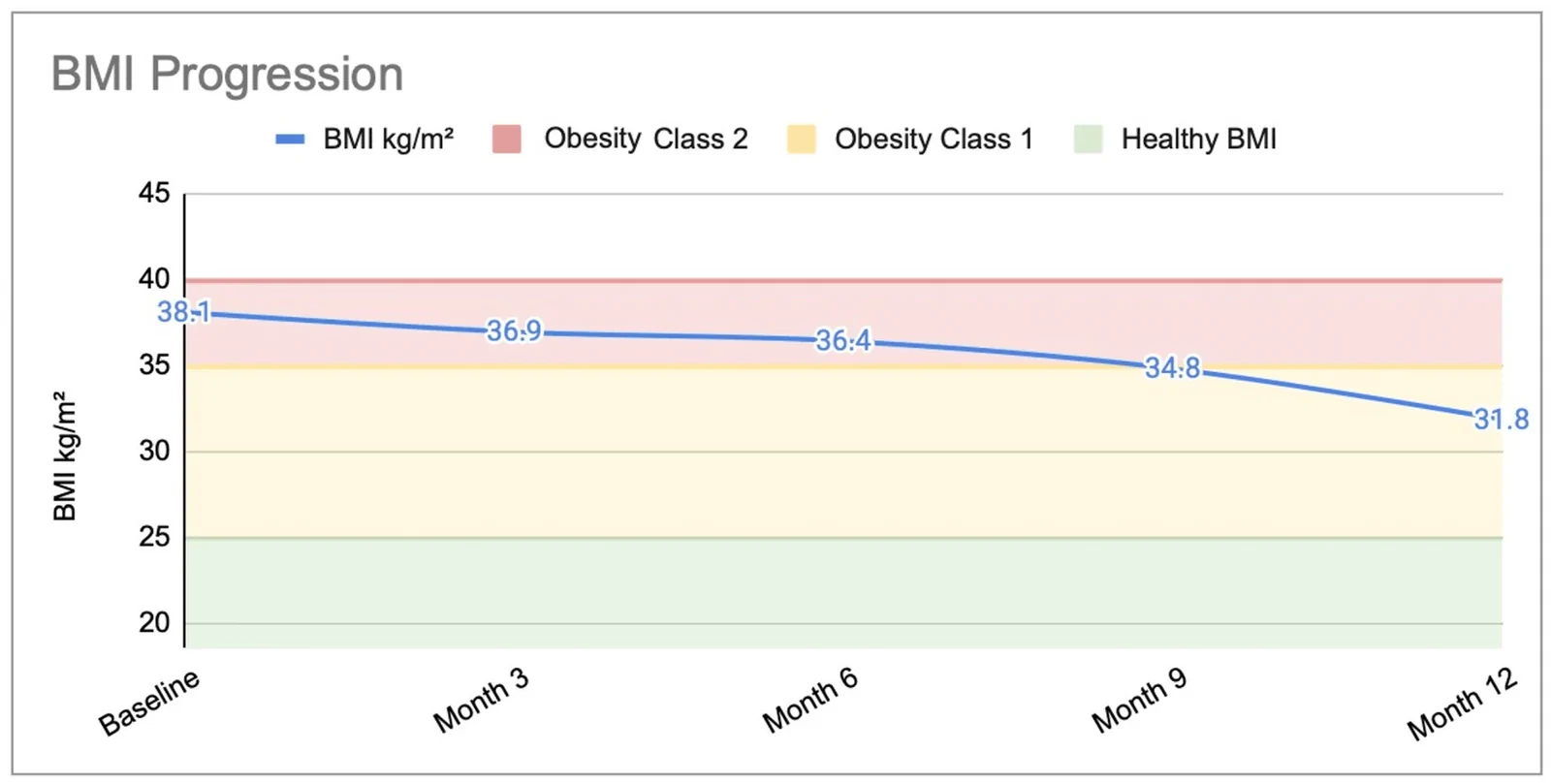
Progression of body mass index (BMI) over the monitoring period

## Discussion

One of the most common challenges facing clinicians today is helping their patients achieve long-lasting weight loss and maintain a healthy weight [1,2]. GLP-1 agonists have revolutionized clinical care over the last decade and led to significant changes in obesity management and therapeutic approaches to weight optimization [3]. Benefits of GLP-1 receptor agonists include reducing obesity-related comorbidities, particularly cardiovascular risk [4]. Research shows that lifestyle modification is essential, even in conjunction with the use of medication, to achieve long-term optimal weight [5]. In managing the patient presented in this case, a structured, four-step clinical framework, which functions as an objective clinical checklist, was utilized to evaluate and improve lifestyle habits, alongside the use of medication.

While behavioral modification is challenging, there is a predictability to this habit-creation process. Behavior change must extend beyond simple clinical instruction, as habits only change when the patient demonstrates sufficient commitment and internal motivation [6]. Thus, the critical first step is to evaluate and foster a patient’s readiness to change a given habit and to understand that behavioral readiness is a continuum rather than an all-or-nothing threshold [7]. A patient may demonstrate full or partial readiness to change a given habit or might be resistant toward altering a specific behavior. Accordingly, the clinician should focus on habits the patient is fully ready to change. For example, a patient may be willing to start weight resistance training but not ready to eat a healthier diet [8]. Another patient may be willing to reduce the consumption of processed food but may not be willing to start an exercise program. Therefore, interventions should be individually tailored to the personal circumstances of each patient, and recommended behavioral changes should begin with targets that the patient is willing and able to address [9].

In a clinical setting, it is helpful to guide the patient to stratify lifestyle habits into a three-tiered categorization system. Using this system, the patient is encouraged to assign habits into one of three categories: habits the patient is willing to change, habits the patient is thinking of changing, and habits the patient is not currently willing to address. Clinical interventions should focus on the first category. To maximize the probability of long-term adherence to a weight management program, it is recommended to target a maximum of two targets simultaneously.

Encouraging the patient to select two specific behaviors from the active readiness category enhances the probability of successful compliance with habit change. Clinicians can guide this selection by prompting the patient to identify changes that offer the highest likelihood of success or have the greatest perceived personal importance. The re-evaluation of behavioral goals should occur at each subsequent follow-up visit as the timeline for successful habit formation varies among individuals [6]. Regular monitoring at follow-up visits should focus on patient motivation and barriers to habit adoption and the iterative modification of the treatment plan to reflect successes and challenges.

After assessing commitment and motivation, clinical intervention shifts focus to the four foundational pillars of lifestyle modification. These interdependent domains combine to support overall metabolic health and sustainable weight loss. The four essential categories are nutrition, physical activity, sleep, and stress management [10].

The first component of this framework for a healthy lifestyle is proper nutrition. One main cause of the current obesity crisis is that healthy, nutrient-dense, real food has largely been replaced by ultra-processed, high-calorie, nutrient-deficient products [1,11-13]. Highly processed foods increase appetite and increase food cravings while providing minimal nutritional value, which makes weight maintenance challenging [3]. This combination leads patients to overeat, creating a positive calorie balance and progressive weight gain [2,3]. Thus, the first step in reaching an optimal weight requires a shift from ultra-processed, high-calorie foods to a whole-food diet, such as fruits, vegetables, healthy oils, and lean protein, mirroring the elements of the Mediterranean diet [3,8,13]. For the patient presented in this case, the whole-food approach is particularly important as eliminating caloric beverages and highly processed sodium-dense foods lowers systemic inflammation and assists in blood pressure regulation, which mitigates the risks associated with his paroxysmal atrial fibrillation.

As it is impractical for a patient to completely avoid packaged foods, this patient was advised to use digital barcode-scanning software to enhance his awareness of the nutritional value and processing of packaged foods. In addition, the 57-year-old patient described in this case study was advised to regularly track and monitor his dietary intake, as this practice stands as one of the strongest clinical predictors of long-term weight loss success [11]. Establishing and monitoring objective daily targets for caloric, protein, and fiber intake enabled this patient to perform real-time adjustments to ensure compliance with his weight management program [1,6]. Sufficient protein, fiber, and hydration are extremely important for patients concurrently initiated on a GLP-1, so the clinician should provide specific recommendations to each patient [3,5]. In addition, sufficient fiber intake through incorporating fruits and vegetables and proper hydration prevent treatment-induced constipation [1]. Further, consuming sufficient protein, along with resistance training, is recommended to promote improvements in muscle mass during periods of rapid weight loss, which is a priority for this 57-year-old patient whose advanced knee osteoarthritis requires him to preserve skeletal strength [11].

In addition, this patient was counseled to reduce or eliminate his consumption of alcohol, as alcohol intake introduces empty calories and impairs metabolic efficiency [9]. This patient was also advised to restrict inter-meal snacking to further reduce his exposure to calorie-dense, low-nutrient foods [1,2,8,9,14]. In addition, this patient was encouraged to incorporate a time-restricted eating framework, which can be a useful tool in reducing total daily calorie consumption [14]. To ensure proper nutrition while engaged in a weight loss plan, targeted nutritional supplements should be evaluated on an individualized basis for each patient [6]. For example, whey protein or creatine monohydrate supplementation can be introduced, alongside resistance training, to promote muscle gain and avoid muscle loss, which is a concern in patients using a GLP-1 [6,12,15-17]. Micronutrient supplements, such as folate and vitamin B12, may be indicated for patients exhibiting deficiencies or for those taking medications known to deplete those nutrients [11]. Probiotics, as well, can be helpful in certain patient populations [11]. Supplement choices should be guided using a certified third-party analytical platform so the clinician can ensure that recommended supplements are manufactured under strict quality standards.

This 57-year-old patient reported compliance in reducing the consumption of processed foods and alcohol and incorporating a high-protein, whole-food-based diet.

The second component of the structured lifestyle framework addresses the patient’s physical activity level [16]. Clinically, patient compliance is often best achieved by utilizing the term “activity level” rather than “exercise,” as the latter term can generate psychological resistance. Initial management requires a baseline assessment of the patient’s current activity level, most often measured by current steps per day, which can be easily monitored via the use of a standard smartphone pedometer utility [6,16]. For highly sedentary patients, a structured progression, such as incorporating a 10-20-minute walk after each meal and weekly increases in the daily step count goal by 1,000 steps, provides a sustainable path to achieving 10,000 steps per day [6,16]. Once the 10,000 steps per day milestone is achieved, patients engaging in rapid weight loss facilitated by a GLP-1 receptor agonist benefit from adding resistance training, as recent studies show that building and maintaining muscle mass is one of the best predictors of long-term health and maintenance of an optimal weight [6,16]. As was appropriate for the patient described in this case, ambulatory goals must be individually modified to accommodate each patient’s underlying health conditions [16]. This patient reported increasing his physical activity through swimming and bicycle riding.

Optimal sleep represents the third step in changing habits to support a healthy weight loss program, as suboptimal sleep negatively impacts long-term weight reduction and healthy weight maintenance [17,18]. Accordingly, always consider screening for underlying sleep disorders or referral to a sleep medicine specialist if a concern is suspected [17,18]. In the context of the current patient’s history of severe obstructive sleep apnea, which is already managed by the use of CPAP therapy, behavioral modifications may be beneficial in conjunction with medical therapies.

To improve sleep efficiency, clinicians can introduce a structured, time-based sleep hygiene protocol. Under this framework, this 57-year-old patient was counseled to cease all caffeine consumption at least 10 hours prior to bedtime, discontinue the consumption of food and fluids three hours before sleep, refrain from work or stressful activities two hours before bed, and eliminate electronic screen exposure one hour before retiring. The complete elimination of alcohol is an adjunctive recommendation of this protocol. By transitioning this “10-3-2-1” framework into an objective, clinical narrative, lifestyle methodology remains fully aligned with academic peer-review standards while being easily understood and followed by patients. This patient reported that his sleep hygiene remained suboptimal during the monitoring period.

Structured stress management represents the final foundational pillar of the lifestyle modification framework, which was used in clinic to affect behavioral change in this 57-year-old patient, as modulating the stress response is particularly important when implementing a medical weight reduction protocol [19]. Patients experiencing chronic psychosocial stress are less likely to successfully achieve the sustainable habit change necessary to lose weight [6,9,19]. As stress is an inevitable part of the human experience, clinical focus must be on helping patients learn to mitigate and manage the level of stress in their lives, rather than attempt complete elimination.

One of the most effective clinical tools to help patients self-regulate chronic autonomic arousal is a mindfulness-based intervention, such as meditation [6,9,19]. Meditative practice trains individuals to separate from the stream of thoughts that exacerbate psychological distress [19]. While external events often serve as a catalyst for stress, it is the cognitive analysis and internal processing of these events that trigger stress mechanisms in the body [19]. The ability to objectively observe internal cognitive and emotional states without triggering a biochemical stress reaction differentiates individuals who exhibit high psychological resilience from those susceptible to stress-induced metabolic dysfunction [9,19].

Meditation is one of the most accessible ways to cultivate the neurological skillset that limits the psychological and physiological effects of stress [19]. The meditative state can be achieved through a diverse array of modalities that involve physical or mental repetition, such as mindful walking, breathing exercises, or silent repetition of a mantra [9,19,20]. Effective meditation relies on a two-phase cognitive framework [20]. The initial phase involves the mindful execution of a repetitive focus, such as repeating a mantra. In the subsequent phase, the practitioner is instructed to passively observe a distraction, consciously release it, and then refocus attention on the primary repetition. This repeated cognitive shifting induces a reduction of the biochemical stress reaction [20].

It bears noting that there are clinical challenges that should be considered when using a GLP-1 receptor agonist, especially in a complex patient with atrial fibrillation. Studies have shown that GLPs can be associated with a temporary mild increase in heart rate, typically 2-4 beats per minute [8]. This may be related to a mild sympathetic nervous system stimulation resulting in increased metabolic demand. This effect tends to reduce or resolve over time.

Due to gastrointestinal (GI) overlap, symptom masking or exacerbation should always be considered when caring for patients on GLPs. GLPs, especially at higher doses, can alter the biochemistry and functioning of the GI system, which can mask or trigger serious issues such as gastrointestinal conditions (irritable bowel disease {IBD}, celiac disease, colorectal cancers, etc.) or other conditions such as eating disorders [3,5]. Moreover, GLPs can occasionally be associated with significant side effects such as pancreatitis, gallbladder disease, and gastroparesis [4]. Thus, patients should be monitored carefully, and measures should be taken to minimize these risks, including counseling patients on sufficient hydration and fiber intake, as well as using the lowest effective dose of GLP. Sarcopenia is also a concern with patients on GLPs, as it is for any patient pursuing weight loss through a reduction in caloric intake [3,16]. Sarcopenia is believed to relate to rapid weight loss without a structured strength training program and/or sufficient protein intake with or without taking a GLP [16]. As mentioned previously in this article, all patients on a weight loss program should be counseled on the importance of strength training and protein intake [16].

Another important consideration is the paradox that GLPs partly work by reducing GI motility, leading to a reduction in appetite and calorie consumption, but this GI dysmotility can sometimes lead to difficulty unloading [3,5]. Fortunately, this effect can generally be avoided through proper hydration and fiber intake and using the lowest effective GLP dose. Occasionally, laxatives may be needed in some situations.

Another important consideration is that a reduction in activity, including total steps and moderate to vigorous physical activity, due to mechanical barriers is commonly seen in these patients [16]. This often relates to musculoskeletal disease, most notably osteoarthritis, which is commonly seen in these patients [16]. Exercise is a vitally important component of any lifestyle modification program, so it is imperative that patients be counseled to anticipate this issue [7,9,15]. De-loaded exercises such as swimming, seated resistance band exercises, and recumbent cycling can often be recommended with good success [16]. Shorter sessions, extended warm-up periods, low-impact resistance band exercises, and bodyweight movements can also be helpful to many patients [16]. Smaller meals with longer periods of time between meals can also be helpful. Many of these patients, including the patient in this paper, have obstructive sleep apnea treated with CPAP. GLPs can be helpful in treating obstructive sleep apnea, but it is important to remember that there is a therapeutic lag effect in this treatment, as typically 10%-20% body weight reduction must be achieved before there is a clinically relevant reduction in sleep apnea scores [5]. During this lag period, obstructive sleep apnea can remain clinically active. Thus, GLPs should be considered a complementary agent rather than a primary treatment modality for clinically significant obstructive sleep apnea, and CPAP should not be discontinued without a follow-up sleep evaluation [3].

Patients on GLPs are often on other pharmaceutical agents, so various interactions need to be considered. For example, GLPs can delay gastric emptying, not only delaying peak concentrations but also extending exposures for oral medications, which may necessitate dosage changes [3]. Moreover, combining GLPs with insulin or insulin secretagogues can increase the risk of clinically significant hypoglycemia. Conversely, patients often achieve improved blood pressure, cholesterol, and sugar control while on a GLP, so other medications may need to be adjusted to account for this metabolic improvement [5,6,8].

For the 57-year-old patient described in this case study, suppressing excess sympathetic activation through targeted stress management acted as an important clinical adjunct to the use of a GLP-1 receptor agonist. This practice assists in reducing the cortisol burden, thus helping improve the patient’s hepatic steatosis and visceral obesity. During routine follow-up visits, this patient was taught practices such as box breathing and 4-7-8 meditation and was also encouraged to use validated noncommercial digital mindfulness applications to facilitate daily compliance [19]. This patient self-reported that he practiced recommended stress-reduction techniques on a regular basis.

## Conclusions

This is a case report of a 57-year-old man with a history of morbid obesity, paroxysmal atrial fibrillation, hyperlipidemia, hepatic steatosis, hypogonadism, degenerative joint disease, and obstructive sleep apnea using CPAP who presented for a routine follow-up appointment, where he was started on a GLP-1 receptor agonist. In addition to the medical management of his obesity and concomitant medical issues, the patient was counseled on adopting a behavior modification framework to optimize his chances for long-term success.

The patient’s therapeutic progress was monitored through regular, structured follow-up appointments over a 12-month period to track his weight loss and concomitant medical issues. Monitoring involved quarterly visits supplemented with monthly messaging. During this time frame, regular clinical adjustments were made to the dosage of his GLP-1 receptor agonist to assist in implementing the recommended behavioral modification framework. This case illustrates how the combination of a GLP-1 receptor agonist and adherence to a practical, four-step lifestyle modification framework focusing on the four foundational pillars of nutrition, physical activity, sleep hygiene, and stress management can be efficiently operationalized within routine clinical follow-up encounters.
